# Thyroid surgery volume – A statement issued by the Brazilian Head and Neck Surgery Society (SBCCP)

**DOI:** 10.20945/2359-4292-2024-0064

**Published:** 2024-06-03

**Authors:** Fatima Cristina Mendes de Matos, José Guilherme Vartanian, José Carlos Barauna, Ary Serrano Santos, Achilles Alves de Levy Machado, Paola Andrea Galbiatti Pedruzzi, Murilo Catafesta das Neves, Flavio Carneiro Hojaij, Marianne Yumi Nakai, Aline de Oliveira Ribeiro Viana, Izabella Costa Santos, Rafael de Cicco, Renato de Castro Capuzzo, Fábio de Aquino Capelli, Dorival De Carlucci, Gilberto Vaz Teixeira, Beatriz Cavalheiro, Fabio Brodskin, Ivan Agra, Fernando Luiz Dias, Marco Aurélio Kulcsar, Giulianno Molina de Melo

**Affiliations:** 1 Sociedade Brasileira de Cirurgia de Cabeça e Pescoço São Paulo SP Brasil Sociedade Brasileira de Cirurgia de Cabeça e Pescoço (SBCCP), São Paulo, SP, Brasil; 2 Instituto Nacional de Câncer Rio de Janeiro RJ Brasil Instituto Nacional de Câncer (INCA), Rio de Janeiro, RJ, Brasil; 3 Universidade de São Paulo Instituto do Câncer do Estado de São Paulo São Paulo SP Brasil Instituto do Câncer do Estado de São Paulo (Icesp), Universidade de São Paulo (USP), São Paulo, SP, Brasil

**DEAR EDITOR AND COLLEAGUES,**

Concerning the article "Treatment strategies for low-risk papillary thyroid carcinoma", published in the Archives of Endocrinology and Metabolism, the Brazilian Society of Head and Neck Surgery (SBCCP) has received with great concern the following statement: "In our opinion, either [partial thyroidectomy] or lobectomy is a good option for patients in Brazil, especially considering the limited number of head and neck surgeons with high surgical volume in the country" (1).

According to recent data from the Brazilian National Cancer Institute (INCA), thyroid cancer has an estimated yearly incidence of 16,600 cases and is the fifth cause of cancer in the female population in Brazil (2). Given this high incidence, along with the increased prevalence of advanced benign thyroid diseases in the Brazilian population and the fact that thyroidectomy is the most common and available treatment option for these conditions, it is important to highlight the following key points related to recent (2022) Brazilian data:

- 22,780 surgical procedures related to the thyroid were performed within the private health care system (3).
- 18,945 thyroidectomies were performed within the Unified Care System (*Sistema Único de Saúde* – SUS) (4).
- 41,725 thyroid procedures were performed in total within both public and private health care systems.

Training of head and neck surgeons usually involves a high volume of thyroid surgeries. According to 2022 data from A.C. Camargo Cancer Center, INCA, and the University of São Paulo-Icesp, each head and neck surgery resident at these institutions performs yearly about 160, 60, and 50 thyroid surgeries, respectively (data presented in the 2023 Annual Meeting of the American Academy of Otolaryngology-Head and Neck Surgery, TN, USA).

We want to emphasize that SBCCP is the second largest society of head and neck surgery in the world and is distinguished not only by its membership size but also by its recognized quality of care, as acknowledged by leading global centers. Currently, SBCCP has 792 active members. Some members are dedicated to the treatment of squamous cell head and neck carcinomas of the upper aerodigestive tract, skin cancers, and thyroid diseases, while others focus only on thyroid surgeries. However, most members are experts in the thyroid area. Although the compilation of surgical data is challenging, particularly in cases of uninsured surgeries, the average number of thyroidectomies performed yearly by each SBCCP member is around 53. Based on publications indicating that a high-volume surgeon performs 30–50 thyroidectomies yearly, we can conclude that the SBCCP members are considered high-volume thyroid surgeons (5).

Only with accurate data can we implement targeted health policies to allow equal health care access to our population. The importance of precise reporting, particularly in open-access scientific publications, cannot be overstated, as inaccuracies can adversely affect perceptions and decisions regarding medical procedures.

Our medical specialties must collaborate closely, enhancing health care delivery and treatment options, with the ultimate goal of prioritizing and improving patient care.
